# Assessing the Effect of Riluzole on Motor Unit Discharge Properties

**DOI:** 10.3390/brainsci14111053

**Published:** 2024-10-24

**Authors:** Ehsan Shandiz, Gabriel Lima Fernandes, Joao Saldanha Henkin, Pamela Ann McCombe, Gabriel Siqueira Trajano, Robert David Henderson

**Affiliations:** 1 Department of Neurology, Toowoomba Base Hospital, Toowoomba, QLD 4350, Australia; ehsan.shandiz@health.qld.gov.au; 2 School of Exercise & Nutrition Sciences, Queensland University of Technology, Brisbane, QLD 4000, Australia; g2.fernandes@qut.edu.au (G.L.F.); joao.saldanhahenkin@qut.edu.au (J.S.H.); g.trajano@qut.edu.au (G.S.T.); 3 Centre for Clinical Research, University of Queensland, Brisbane, QLD 4006, Australia; pamela.mccombe@uq.edu.au; 4 Department of Neurology, Royal Brisbane & Women’s Hospital, Herston, QLD 4006, Australia

**Keywords:** ALS, MND, multi-channel surface EMG, HDsEMG, riluzole, electromyography

## Abstract

**Background.** This study aims to determine if Riluzole usage can change the function and excitability of motor neurons. **Methods.** The clinical data and indices of motor neuron excitability were assessed using high-density surface EMG parameters from 80 ALS participants. The persistent inward current was assessed using the discharge rate from paired motor units obtained from the tibialis anterior muscle. This enabled the discharge rate at recruitment, peak discharge rates and the hysteresis of the recruitment–derecruitment frequencies (also known as delta F) to be calculated. Limbs were classified according to their strength. **Results.** No differences in these motor neuron discharge properties were found according to whether Riluzole was used. **Conclusions.** The possible interpretations of this finding are discussed.

## 1. Introduction

Neuronal hyperexcitability and glutamate toxicity have been proposed as a pathogenic mechanism in amyotrophic lateral sclerosis (ALS) [1]. Riluzole (2-amino-6-trifluoromethoxybenzothiazole) is a sodium channel inhibitor that particularly inhibits persistent sodium currents with a reduction in the release of the neurotransmitter glutamate and increase in the uptake of extracellular glutamate by astrocytes, leading to a reduction in toxicity to neurons [2]. In slice preparations from a mouse model study of ALS, Riluzole effectively suppressed the frequency of repetitive discharge of lower motor neurons in response to the injection of current, and this effect was associated with the drug’s ability to inhibit both glutamate release and sodium channel activity [3]; however, Riluzole has not consistently demonstrated therapeutic efficacy in several experimental animal models of ALS [4,5,6,7,8,9]. Riluzole reduced the progression of ALS in two major clinical trials [10,11], and since then has been widely approved for use. Recent reviews favour a survival advantage for those taking Riluzole [12]. Despite the efficacy of Riluzole in prolonging the survival of ALS patients, there remains uncertainty about when in the disease stage benefit occurs from Riluzole usage [13]. Riluzole leads to transient modulation of cortical and axonal hyperexcitability in ALS patients [14], suggesting that the drug may not permanently alter the underlying hyperexcitability in the disease.

High-density surface electromyography (HDs-EMG) is a powerful, non-invasive tool to evaluate the discharge characteristics of motor neurons. It allows the study of a variety of indices of motor neuron function including the discharge rate at recruitment, peak discharge rates and the hysteresis of the recruitment–derecruitment frequencies (i.e., Δ frequency, or ΔF). Paired motor unit recordings, allowing for ΔF evaluation, represent a non-invasive methodology for estimating the intrinsic motoneuron excitability in humans in vivo, which is a potential biomarker for ALS [15,16,17,18]. We have previously found that, in participants with ALS, there are changes in these parameters as the disease progresses [19].

The aim of the present study was to determine if Riluzole usage can change the function and excitability of motor neurons by assessing the clinical data and indices of motor neuron excitability using HDs-EMG in ALS participants. We hypothesised that Riluzole can alter motor neuron excitability in people living with ALS, and that the changes could be affected by the disease stage.

## 2. Methods

### 2.1. Participants

This study was conducted to assess the effects of Riluzole on motor neuron function in ALS participants using HDs-EMG and clinical assessments. Ethical approval was obtained from the Royal Brisbane and Women’s Hospital-Metro North Office of Research (Human Research Ethics Committee number HREC/2022/QRBW/81715). All participants provided written consent.

A total of eighty participants diagnosed with ALS based on the 2019 Gold Coast criteria were recruited for this study [20]. Most patients seen through the multi-disciplinary ALS clinic in 2022–2023 were screened and offered inclusion in the study. In order to consider the wide applicability of HDs-EMG, patients were only excluded if no visible movement of foot dorsiflexion could be seen from either side. Participants were divided into two groups: those who were on Riluzole treatment (Riluzole users) and those who were not (non-Riluzole group). Riluzole as a therapy was prescribed at diagnosis and continued to the recruitment date. In Australia, concern with the efficacy and side effect profile of Riluzole has led to a low uptake of this drug. The Medical Research Council’s scale (MRC scale) was administered by an experienced neurologist (ES). The tibialis anterior muscle was chosen as a practical, commonly involved muscle in ALS. Both tibialis anterior muscles from the lower limb were examined and the score was used to classify each limb muscle as strong (MRC score of 5) or weak (MRC less than 5), and this terminology was used for the clinical assessment, recognising that stronger limb muscles likely already have evolving motor neuron pathology, as can be seen with EMG. As previously reported, a participant could have both lower limb muscles within the same group [19]. The revised functional rating scale (ALSFRS-R) was recorded by ES to be used as clinical data in this study [21].

### 2.2. High-Density Surface Electromyography (HDs-EMG) Assessment

The tibialis anterior muscle was used for all studies. High-density surface electromyography (HDs-EMG) was recorded during submaximal ramped-up (triangular) contractions targeting 40% of their maximal root-mean-squared (RMS) EMG value [19]. Surface electromyography signals were recorded using 64-channel electrode grids (GR08MM1305, OTBioelettronica, Torino, Italy). The electrode was positioned following the estimated muscle fibre orientation, using a bi-adhesive layer with conductive paste to ensure good skin–electrode contact and conductibility. A ground strap electrode (WS2, OTBioelettronica, Torino, Italy) was dampened and placed around the ankle joint of the tested leg. The HDs-EMG signals were acquired in monopolar mode, amplified (256×), band-pass-filtered (10–500 Hz), and converted to digital signals at 2048 Hz by a 16-bit wireless amplifier (Sessantaquattro, OTBioelettronica, Torino, Italy), before being stored for offline analysis. HDs-EMG signals were recorded and analysed offline, and decomposed into motor unit spike trains with specialised software using a blind source separation decomposition technique, DEMUSE tool software (v.4.1; The University of Maribor, Slovenia) [22].

### 2.3. Experimental Protocol

**Figure 1:**
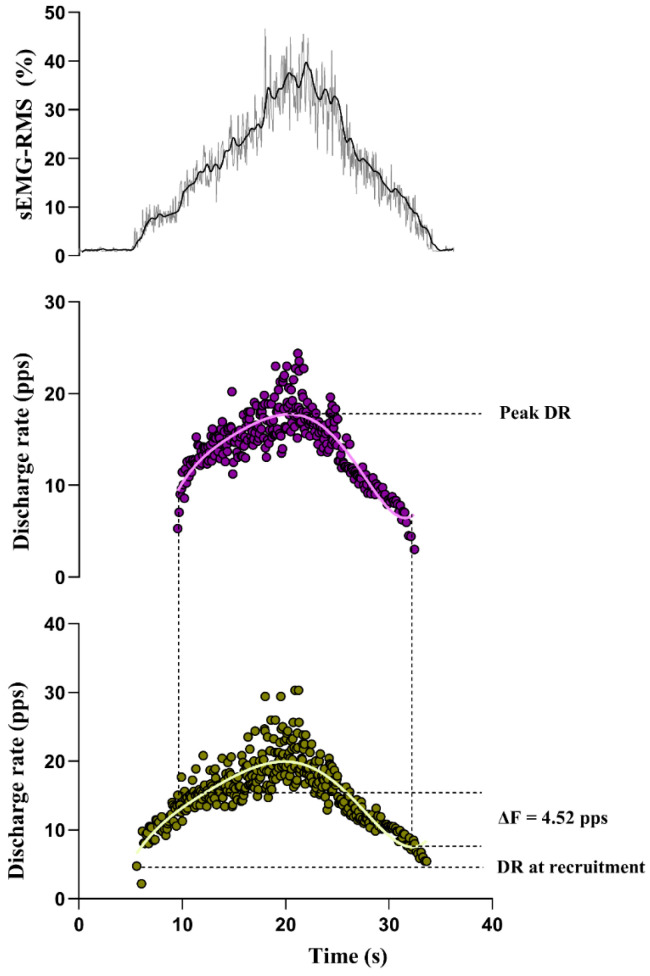
Illustration of ΔF calculation. **Top** panel displays a triangular contraction to 40% of the participant’s sEMG-RMS MVIC. The **middle** and **bottom** panels illustrate ΔF calculations. The test motor unit (top, purple) and control motor unit (bottom, yellow) are shown. ΔF is calculated as the difference between the discharge rates of the control unit at the recruitment and derecruitment times of the test unit. A 5th-order polynomial fit (light purple and light-yellow lines over the motor units) was created from the instantaneous discharge rates of each motor unit. Peak DR was considered the maximum value obtained from the polynomial curve, and the instantaneous rate between the two initial discharges was considered the DR at recruitment. DR—discharge rate; MVIC—maximal voluntary contraction; sEMG—surface electromyography; RMS—root mean square; pps—pulses per second.

Participants were positioned sitting on a chair with their knees bent at 90° flexion, their ankles in an anatomical position, and their feet touching the ground. Participants then performed two maximal isometric ankle dorsiflexion contractions (MVICs). After that, participants performed two triangular contractions to 40% of their peak sEMG-RMS, obtained from their MVIC, at a speed of 15 s up and 15 s down. Participants received visual feedback during each contraction and were instructed to follow the path displayed on a 14-inch laptop monitor positioned in front of them. The order in which each side was tested was randomised. If no motor function or sEMG signal could be detected for a specific side due to disease progression, the limb muscle was not tested. The single best triangular contraction, with the lowest deviation from the sEMG RMS-trace trajectory, was analysed. All motor units were manually inspected by a trained investigator. Only motor units with a pulse-to-noise ratio > 30 dB and sensitivity > 90% were considered for data analysis [23]. The discharge events for each motor unit were converted into instantaneous discharge rates and fitted into a fifth-order polynomial. Persistent inward current (PIC) amplitude was estimated using the paired motor unit analysis [24]. Motor units with a lower recruitment threshold (control motor units) were paired with others with higher recruitment threshold (test motor units). ΔF was calculated as the change in discharge rates (DRs) of the control motor unit in the time between recruitment to the derecruitment of the test motor unit [24,25]. (Figure 1) ΔFs obtained for each control motor unit were averaged to obtain a single ΔF for each test motor unit. The maximum value obtained from the polynomial curve was considered the peak motor unit discharge rate (DR), and the instantaneous rate between the two initial discharges was considered the DR at recruitment (see (Trajano et al., 2023) for more details) [19].

### 2.4. Statistical Analysis

Separate linear mixed-effect models were used to compare ΔF, peak discharge rate (DR), and DR at recruitment with limb muscle strength (strong and weak) and Riluzole use. Peak discharge rates have been previously shown to influence ΔF; thus, peak DR was included in the ΔF model as a covariate. A random intercept and slope (limb side, i.e., right or left) were included for each participant in the study to account for the correlation between repeated observations. All statistical analyses were conducted in RStudio (version 2023.09.1 + 494). A significant difference was accepted at *p* ≤ 0.05. The estimated marginal mean difference and 95% confidence intervals (CI) for ΔF, peak DR, and DR at recruitment were determined using the emmeans package [26]. Data are reported as means (±95% CI lower and upper limits), unless stated otherwise.

## 3. Results

In total, 80 participants with ALS (63 ± 11.7 years old, 52 males,) volunteered for this study. Of these, thirty-two participants had been on Riluzole since diagnosis and up to the period of data collection (Table 1). Clinical indices including mean ALSFRS-R and MRC scores were not significantly different between Riluzole users and non-users (Table 2). Mean disease duration, classified as time (months) from symptom onset to date of data collection, was 31.52 (±27.97) for the total group, 34.81 (±33.11) for the Riluzole treatment group, and 29.25 (±24.97) for the no Riluzole group.

**Figure 2:**
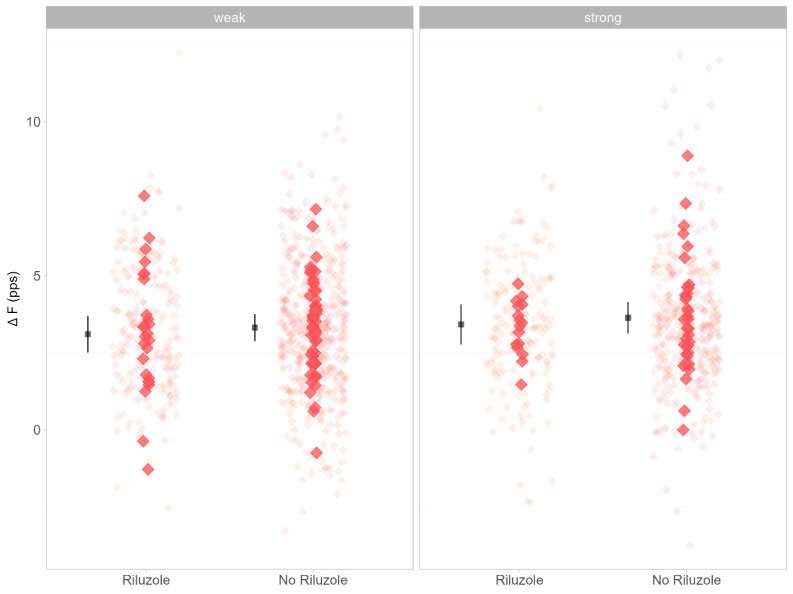
Data represent ΔF for Riluzole and no Riluzole groups. Individual data points are jittered in the background as lighter red diamond. Participants’ mean ΔF values are plotted as darker red diamonds at the front. Estimated marginal mean and 95% confidence interval (CI) are offset to the left. Data are faceted by MRC classification of limb muscle strength (i.e., strong, MRC = 5; weak, MRC < 5).

**Figure 3:**
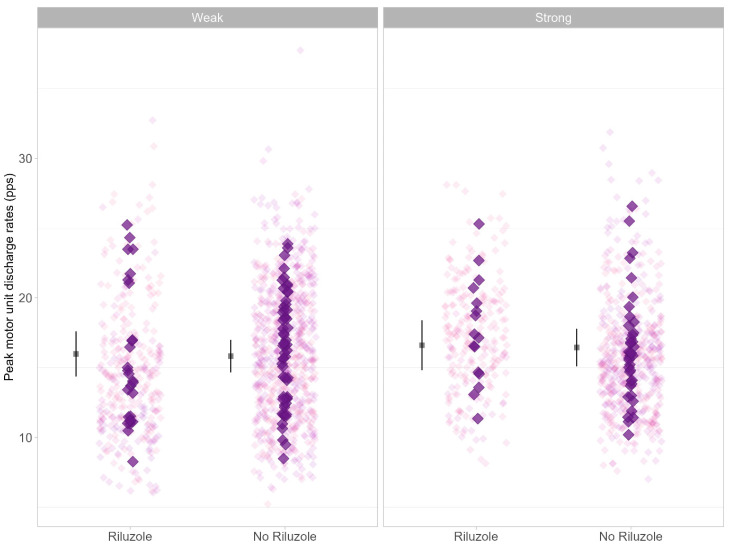
Data represent peak motor unit discharge rate (DR) for Riluzole and no Riluzole groups. Individual data points are jittered in the background as lighter purple diamonds. Participants’ mean peak DRs are plotted as darker purple diamonds at the front. Estimated marginal mean and 95% confidence interval (CI) are offset to the left. Data are faceted by MRC classification of limb muscle strength (i.e., strong, MRC = 5; weak, MRC < 5).

**Figure 4:**
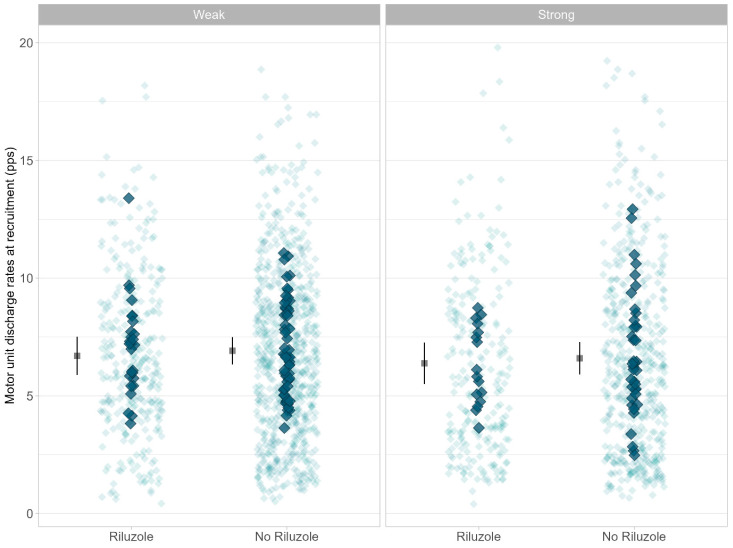
Data represent motor unit discharge rates at recruitment for Riluzole and no Riluzole groups. Individual data points are jittered in the background as lighter blue diamonds. Participants’ mean discharge rates at recruitment are plotted as darker blue diamonds at the front. Estimated marginal mean and 95% confidence interval (CI) are offset to the left. Data are faceted by MRC classification of limb muscle strength (i.e., strong, MRC = 5; weak, MRC < 5).

A total of 1755 motor units (15.9 ± 4.5 SD per participant) were recorded, with 1207 motor units yielding paired delta frequency analysis (ΔF). Estimated marginal means are presented in Table 3. ΔF (F = 1.0.30, *p* = 0.312, Figure 2) was not different between weak and strong limb muscles. There was also no difference in ΔF between the Riluzole and non-Riluzole groups (F = 0.059, *p* = 0.808). There was no difference according to sex (F = 2.969, *p* = 0.089). Peak discharge rate (F = 0.404, *p* = 0.525, Figure 3) showed no difference between the Riluzole and non-Riluzole groups, and there was no significant effect of Riluzole (F = 0.017, *p* = 0.894) or sex (F = 0.446, *p* = 0.506). Discharge rates at recruitment (F = 0.474, *p* = 0.492, Figure 4) were also similar between limb muscles, with no significance influence of Riluzole (F = 0.189, *p* = 0.664) or sex (F = 0.696, *p* = 0.407).

## 4. Discussion

This study used high-density surface electromyography (HDs-EMG) to study the excitability of lower motor neurons supplying the tibialis anterior muscle of 80 participants with ALS, and to compare motor unit properties between those on Riluzole and those who were not on Riluzole. The motor unit properties that were evaluated using HDs-EMG were ΔF, peak motor unit discharge rates, and motor unit discharge rate at recruitment. These properties allow a measure of lower motor neuron excitability [15,27] and are governed both by descending upper motor neuron pathways and also by descending modulatory pathways [28,29]. Using HD-sEMG to understand the role of these modulatory pathways in ALS is an emerging field of interest [16,17,18]. This study was performed to investigate whether Riluzole, which has an overall clinical benefit for survival [10,11,12], affects these parameters and whether there is an effect at different disease stages. We did not observe differences in any of the motor neuron properties from HDs-EMG according to Riluzole status. There are different possible interpretations for this result—(a) Riluzole may have an early effect, possibly due to neuroprotection from excitotoxicity, or a late effect, possibly on respiratory function [30,31]. Using axon excitability (within a larger study), Riluzole therapy led to a transient increase at four weeks in the relative refractory period, superexcitability, and late subexcitability, all of which returned to baseline levels eight weeks after initiation of Riluzole [14]. (b) The predominant effect of Riluzole is on the central motor neuron pathways and not well translated to nerve/muscle, as demonstrated by electrical impedance myography in a G93A mouse model [32] and in studies combining cortical and peripheral studies [29]. In contrast, a meta-analysis of Riluzole use showed that significant changes in bulbar and limb function occurred independent of muscle strength [13], which may help explain the inability to show differences between the groups in our sample.

### Limitations

The effect of Riluzole may be modest, with participants potentially using Riluzole for different periods of time, and this study may not have had sufficient power to detect a difference. The designation of muscle strength was made by clinical assessment (within the limitations of the MRC grading) and EMG would have allowed a better assessment of denervation. Combining HDs-EMG with TMS would have allowed a better assessment of the motor neuron pathway from the motor cortex to muscle. Against these points is that there was no suggestion of benefit to warrant further exploration using methods assessing peripheral motor pathways. Longitudinal studies assessing changes over time would strengthen the assessment of Riluzole’s effect, particularly studying from the time of commencement of Riluzole. Recruiting larger sample sizes with detailed exposure time to Riluzole would allow us to account for subgroup analysis of different clinical parameters.

## 5. Conclusions

In this study, which investigated data from HDs-EMG of ALS participants at a mid-stage of disease, we found no effect of Riluzole on the motor neuron discharge properties from the tibialis anterior muscle. Further investigation into Riluzole’s mechanisms of action on motor neuron excitability is warranted to determine whether it offers additional benefits for people living with ALS.

## Figures and Tables

**Table 1:** Participant characteristics.

Total Number of Participants (n)	80
Sex	52 males, 28 females
Total limbs tested (i.e., right, left)	143
Weak limbs (MRC < 5)	87
Participants on Riluzole	32
Number of limbs (MRC < 5) on Riluzole/no Riluzole	36/51
Number of limbs (MRC = 5) on Riluzole/no Riluzole	18/38
Mean ALSFRS-R score	34.5 (31.2–35.8)

ALSFRS-R—revised amyotrophic lateral sclerosis functional rating scale. ALSFSR-R—score reported as mean (95% confidence interval).

**Table 2:** Mean ALSFRS score and mean MRC score of recruited participants.

	Riluzole	No Riluzole
ALSFRS-R	34 (±9.9)	35.6 (±7.2)
ALSFRS-R item 8	2.6 (±1.0)	2.9 (±0.8)
ALSFRS-R item 9	1.9 (±1.4)	2.1 (±1.4)
Mean MRC score (weak limb muscle group)	3.3 (±1.2)	3.3 (±1.0)
Disease duration (months)	29.5 (±27.8)	31.4 (±28.3)
Site of onset		
Bulbar	7	13
Upper limb	8	13
Lower limb	9	20

ALSFRS-R—revised amyotrophic lateral sclerosis functional rating scale; ALSFRS-R—item 8, refers to walking; ALSFRS-R—item 9, refers to climbing stairs; MRC, The Medical Research Council’s Scale for muscle strength. Data for ALSFRS-R and MRC scores are presented as means (±SD).

**Table 3:** Estimated marginal means for ΔF analysis, motor unit peak discharge rates (DRs), discharge rates (DRs) at recruitment for weak and strong limb muscles, by treatment group (i.e., Riluzole, no Riluzole).

	Riluzole		No Riluzole	
	Weak Muscle	Strong Muscle	Weak Muscle	Strong Muscle
ΔF (pps)	3.38 (2.93–3.83)	3.64 (2.89–4.24)	3.38 (2.93–3.83)	3.56 (2.89–4.24)
Peak DR (pps)	15.9 (14.8–17.2)	16.3 (14.4–8.2)	16.0 (14.8–17.2)	16.5 (14.4–18.2)
DR at recruitment (pps)	6.69 (5.82–7.56)	6.44 (5.49–7.39)	6.90 (6.28–7.52)	6.65 (5.92–7.38)

ΔF—delta frequency; DR—discharge rate; pps—pulses per second. Data presented as means (95% CI).

## Data Availability

The original contributions presented in the study are included in the article, further inquiries can be directed to the corresponding author.
